# Alexithymia in Patients with Ménière Disease: A Possible Role on Anxiety and Depression

**DOI:** 10.3390/audiolres11010008

**Published:** 2021-02-23

**Authors:** Roberto Teggi, Claudia Yvonne Finocchiaro, Claudio Ruggieri, Omar Gatti, Federica Rosolen, Mario Bussi, Lucio Sarno

**Affiliations:** 1 ENT Division, Department of ENT, IRCCS San Raffaele Hospital, 20132 Milan, Italy; gatti.omar@hsr.it (O.G.); bussi.mario@hsr.it (M.B.); 2 Clinical Healt Psychology Unit, IRCCS San Raffaele Hospital, 20132 Milan, Italy; claudia.finocchiaro@gmail.com (C.Y.F.); ruggieri.claudio@hsr.it (C.R.); rosolen.federica@hsr.it (F.R.); sarno.lucio@hsr.it (L.S.); 3 Faculty of Psychology, Vita-Salute San Raffaele University, 20132 Milan, Italy

**Keywords:** Menière’s disease, alexithymia, anxiety, coping strategies, depression, quality of life

## Abstract

The aim of this paper was to investigate the role of the psychological variable of alexithymia both as a risk factor for the development of Ménière’s disease (MD) and as a component that influences the personal experience of MD and the individual quality of life. We collected data from 179 Italian patients who fulfilled criteria for definite MD. Patients filled out validated self-rating questionnaires to assess alexithymia (TAS-20), quality of life (WHOQOL-BREF), anxiety and depression (HADS), perception of stress (PSS) and coping strategies (COPE). Socio-demographic data and MD clinical features were collected using a specific rating form. Subjects affected by MD showed higher levels of alexithymia compared to general population. Among MD patients, those characterized by high levels of alexithymia revealed a significant increase in anxiety and depression, greater perceived stress, a lower quality of life in psychological health and social relationships domains and the use of less mature coping strategies in comparison with MD patients with low or absent alexithymia. Our preliminary data could help in hypothesizing a role of psychological functioning in MD development and in the adaptation to the disease. The presence of alexithymia in patients suffering from MD may constitute a risk factor for the development of anxiety and depression symptoms; greater perceived stress and for poorer psychological and relational quality of life. Therefore, our study design did not allow causal inferences and further studies are needed.

## 1. Introduction

Ménière’s disease (MD) is an inner ear disorder characterized by episodic attacks of severe vertigo accompanied by ear fullness, hearing loss and tinnitus [1]. Vertigo spells are characterized by rotatory spinning sensation that last from 20 min to 12 h with autonomic symptoms (such as nausea and vomiting), preceded or accompanied by cochlear symptoms (usually unilateral), specifically, hearing loss, sensation of aural fullness and tinnitus. The disease was firstly described by Prosper Ménière in 1861; the commonly accepted pathophysiological mechanism relies on an increased volume of endolymphatic space (hydrops), while etiology of the disorder is still under debate and include genetic autoimmune causal factors; MD also presents an overlapping with migraine [2,3,4]. Diagnostic criteria of MD have been recently established by the Barany Society Committee and are the following [5]:

### 1.1. Definite MD

A. Two or more spontaneous episodes of vertigo each lasting 20 min to 12 h;
B. Audiometrically documented low- to medium-frequency sensorineural hearing loss in one ear, defining the affected ear on at least one occasion before, during or after one of the episodes of vertigo;
C. Fluctuating aural symptoms (hearing, tinnitus or fullness) in the affected ear;
D. Not better accounted for by another vestibular diagnosis.

### 1.2. Probable MD

A. Two or more episodes of vertigo or dizziness, each lasting 20 min to 24 h;
B. Fluctuating aural symptoms (hearing, tinnitus or fullness) in the affected ear;
C. Not better accounted for by another vestibular diagnosis.

MD is a chronic disease that leads to a progressive hearing loss with tinnitus and repeated unexpected vertigo attacks with unknown frequency and variable residual chronic dizziness.

The initial fluctuating symptoms, with variable and unexpected spells, and the subsequent chronic state are frequently associated with anxiety and depression.

Many types of vertigo disorders provoke anxiety, panic disorders and avoidance behaviors, as confirmed by epidemiological data [6].

Recent investigations have explored whether patients with different organic vertigo syndromes exhibit different psychological features [7]. Overall, patients with MD demonstrated higher levels of psychological disorders, related to the characteristics of vestibular and cochlear symptoms, particularly the unpredictable nature of the illness; they might develop anxiety because of the frequent and unexpected vertigo spells and depression due to the worsening of quality of life [8].

As originally defined by Nemiah et al. [9], alexithymia is a multifaceted construct encompassing difficulty in identifying subjective emotional feelings and distinguishing between feelings and the bodily sensations of emotional arousal, difficulty in describing feelings to other people, an impoverished fantasy life and a stimulus-bound, externally oriented cognitive style. Currently, alexithymia is not considered a discrete disorder, but rather a personality characteristic that is expressed with variable intensity in the general population [10].

Recent research has suggested that alexithymia can be considered as one of several possible risk factors for a variety of medical and psychiatric disorders, as it may increase the susceptibility to the development of a variety of diseases in addition to genetic determinants and emotional stress [11]. High prevalence rates of alexithymia have been identified in patients with a variety of health problems [12], but it has never been investigated in Ménière’s disease.

## 2. Materials and Methods

### 2.1. Sample

We conducted the present study on 179 Italian patients, recruited on a voluntary base. The sample included adults who joined the AMMI (Associazione Malati di Ménière Insieme) and they had a diagnosis of definite MD performed by a senior ENT specialist. All participants should fulfil criteria for definite MD. They received the information about the aim of the study, signed a written informed consent form and completed an online survey. The study protocol was approved by the institutional ethical committee. Inclusion criteria were the following: age ≥ 18, ability to complete self-reporting questionnaires and diagnosis of MD.

### 2.2. Questionnaires

In the first part, we collected socio-demographic data using a specific rating form, including variables as gender, age, marital status and education. Among clinical data, the age of onset of vertigo and the number of attacks in the last year were saved. Then, we asked the participants to complete some self-rating questionnaires measuring different psychological constructs, specifically alexithymia, quality of life, anxiety, depression, stress and coping strategies.

For alexithymia we used the Italian validated version of the Toronto Alexithymia Scale-20 (TAS-20) [13,14], the most frequently used questionnaire for measuring alexithymia. TAS-20 is a 20-item self-report scale with a three-factor structure congruent with the theoretical construct: difficulty in identifying feelings (DIF), difficulty in describing feelings (DFF) and externally oriented thinking (EOT). Each item is scored on a five-point Likert scale ranging from 1 (strongly disagree) to 5 (strongly agree). The TAS-20 scores range from 20 to 100: subjects scoring 61 or more have been suggested to be alexithymic, whereas those scoring 51 or less are considered to be non-alexithymic. Subjects obtaining a score between 52 and 60 have been suggested to be intermediate alexithymic. The scale demonstrated good psychometric properties in validity and reliability; it has been translated in different languages and validated in many different contexts, countries and cultures. In the present work, Cronbach’s Alpha coefficients have revealed good internal consistency both of the scale and the sub-scales.

We assessed the quality of life using the brief version of the World Health Organization Quality of Life instrument (WHOQOL-BREF) [15], which is a 26-item self-report measure developed by the WHOQOL project across a number of centers worldwide. It assesses the individual perceptions in the context of their culture and value systems, and their personal goals, standards and concerns. It measures the following broad domains: physical health, psychological health, social relationships, and environment. Each item is rated on a five-point Likert scale (1–5). WHOQOL-BREF has demonstrated good psychometric properties. It has been translated and validated in many different languages, confirming its reliability in assessing the quality of life. In our study, it has shown good internal consistency through Cronbach’s Alpha analysis.

The Hospital Anxiety and Depression Scale (HADS) [16] is a simple self-report measure to evaluate anxiety and depression in the setting of medical practice. It consists of 14 items, seven reflecting anxiety and seven reflecting depression. Each item is rated on a four-point Likert scale (0–3). The scores range from 0 to 21 for anxiety and from 0 to 21 for depression. The two subscales, anxiety and depression, are independent measures. The HADS has proven reliability and validity [17]. In the present research, it has demonstrated good internal consistency.

The Perceived Stress Scale (PSS) [18] is one of the most widely used psychological tool for measuring the perception of stress [19]. It is a 10-item self-report instrument and aims at exploring perception of stressful experiences related to events and situations over the previous month. Each item assesses feelings and thoughts during the last month and how often subjects felt a certain way. PSS scores have been correlated with biomarkers of stress, such as cortisol [20,21], showing reliable correlations. PSS has been translated in many languages and we used the Italian version [22]. In our study, the scale demonstrated a good internal consistency through Cronbach’s Alpha analysis.

The Coping Orientation to Problems Experienced Inventory (COPE) [23] is a self-report measure developed to assess a broad range of coping strategies and styles of individuals. It comprises 60 items describing different ways people can react in stressful situations. Subjects are asked to rate on a four-point Likert scale ranging from 1 (I usually do not do this at all) to 4 (I usually do this a lot) how often they respond in a certain manner when they are stressed. The inventory includes 15 different strategies, some more and some less functional, each scored from 4 to 16. The test has proven good psychometric properties [23]. The Cronbach’s Alpha coefficients of the sub-scales have shown sufficient reliability. In the present work, we employed the Italian version [24].

### 2.3. Statistical Analyses

Data analyses were performed using SPSS statistical software, version 20 (IBM SPSS Statistics for Windows, Version 20.0. Armonk, NY, USA: IBM Corp.). Firstly, we conducted descriptive statistics (frequency distribution for categorical variables, mean and standard deviation for continuously distributed variables), on socio-demographics, Menière syndrome’s features and psychological characteristics (alexithymia, quality of life, anxiety, depression, perceived stress and coping strategies) of the sample. At a second stage, we used *t*-test to compare the sample scores on the alexithymic dimension with the normative data of the Italian population. Moreover, *t*-test analyses were conducted to compare the two sub-samples (alexithymic and non-alexithymic) of the total study sample on the psychological variables, namely quality of life, anxiety, depression perceived stress and coping strategies. Any p-value lower than 0.05 was considered significant.

## 3. Results

### 3.1. Socio-Demographic Characteristics of the Sample

We recruited 179 subjects diagnosed with MD. Sixty-two out of 179 patients were males (34.6%) while 117 females (65.4%). The mean age at inclusion was 48.8 (Standard Deviation 11.5; range 23–78 years).

### 3.2. Features of Ménière Disease

All the involved subjects declared to have been diagnosed with MD by a senior Ear Nose Throat specialist. We explored some features of the disorder: unilateral low-frequencies hearing loss, episodic vertigo spells preceded by ear fullness and tinnitus. All patients declared of having performed a Magnetic Resonance Imaging of the central nervous system, negative for disorders potentially correlated with vertigo attacks. We report the statistics in Table 1.

### 3.3. Psychological Variables of the Sample and Comparison with Normative Data

All the patients completed the self-rating questionnaires presented above. Table 2 summarizes the results.

We compared TAS-20 total and sub-scales scores with Italian population normative data by *t*-test analysis. The results showed statistically significant differences in the TAS-20 total score above all in the sub-scales “difficulty in identifying feeling” (DIF) and “externally oriented thinking” (EOT): the study sample had significantly higher scores in those dimensions (Table 3).

### 3.4. Division of the Sample According to the Threshold for Alexithymia

Finally we divided the sample according to the threshold for alexithymia identified by the TAS-20 authors [13]. According to the alexithymia threshold, 74 patients (41.3%) were classified as non-alexithymic and 105 (58.7%) as alexithymic.

**Figure 1:**
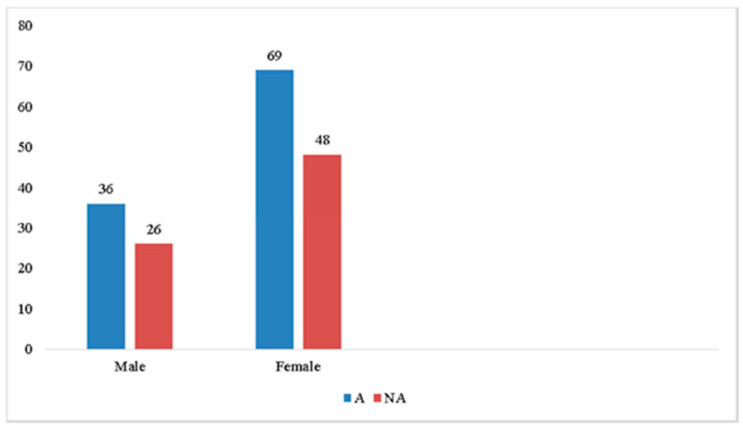
Gender comparison between A (Alexithymic) and NA (Non Alexithymic) groups by Pearson chi-square test.

Moreover, we tested if the two sub-samples were comparable for age, gender and the MD characteristics (onset age, number of crises in the last year). Pearson chi-square test for gender (Figure 1) and *t*-test for age at inclusion, age of onset and number of crises (Table 4 did not show any significant difference between A and NA groups.

Psychological variables: comparison between alexithymic (A) and non alexithymic (NA) groups.

We used a *t*-test to assess differences in psychological variables between A and NA subjects. The quality of life, as measured by the WHOQOL, was significantly lower in the psychological and social relationships domains for the A compared to the NA patients. Instead, the overall quality of life and general health and the physical health and environment domains did not show any statistical difference between the two groups.

Moreover, in A group we found statistically significant differences in the anxiety and depression dimensions (HADS). Alexithymic subjects displayed significantly higher levels of anxiety and depression than the NA patients did. They also experienced greater levels of stress compared to the NA group. The A group scored significantly higher in total stress, perceived stress in the last week and perceived stress in the last month, as measured by the PSS.

Finally, we observed some significant differences between the two groups in the use of coping mechanisms (COPE Inventory). Alexithymic subjects appeared to use more immature and less adaptive coping strategies than NA subjects did.

Table 5 summarizes the results.

## 4. Discussion

In our opinion, the present work introduces an innovative approach to the study of MD, since it investigates for the first time the psychological variable of alexithymia in MD. Particularly, it aims at exploring the role of emotion regulation both as a risk factor for the development of MD and as a component that influences the personal experience of MD and the individual quality of life. We did not consider alexithymia neither as a pathological syndrome nor as an all-or-none phenomenon, but as a personality trait normally distributed in the general population and as a dimensional continuum about the difficulty in recognizing and communicating emotions. According to several studies [24,25,26], alexithymic characteristics are related to sympathetic overreactivity and impaired immune response. The neuroendocrine and immune response of alexithymics seems to follow the same pattern as in subjects afflicted with chronic stress, whereas distress remains emotionally unnoticed [27]. Moreover, many researchers have found that alexithymia may be considered as a risk factor for various medical and psychiatric disorders [12,28,29,30,31].

The present study highlights, in a sample of subjects affected by MD, some specific features influenced by alexithymia. MD subjects showed higher levels of anxiety and depression compared to general population, as suggested by previous researches [8]. However, MD patients characterized by high levels of alexithymia revealed a significant increase in anxiety and depression in comparison with MD patients with low or absent alexithymia. Moreover, alexithymic MD patients seemed to perceive greater stress, as measured by PSS, and a lower quality of life in psychological health and social relationships domains. Furthermore, non-alexithymic subjects displayed more frequently more mature coping strategies, such as positive reinterpretation and growth, use of instrumental and emotional social support, active coping, humor, acceptance and planning, compared to alexithymics. On the other hand, alexithymic subjects resulted to use preferably less mature coping strategies, like behavioral disengagement.

These results suggest that the presence of alexithymia, in a sample of MD subjects, may constitute a risk factor for the development of anxiety and depression symptoms and greater perceived stress. Therefore, MD seems not to be sufficient to explain the presence of psychological consequences, which might emerge due to the co-presence of MD and stable personality traits connected to emotion regulation and expression.

Further, these preliminary data could help in hypothesizing a role of psychological functioning in MD development and in the adaptation to the disease. As noticed before, emotion regulation and stress management influence the way our organism responds, for example, to inflammatory processes through the immune system. Psychoneuroendocrine immunology has pointed out the existence of interrelations between the environment and the organism’s somatic responses through the mediation of personality stable characteristics linked to the stimuli processing and elaboration [28].

According to different authors, MD may arise from the interaction between familiar predisposition and predisposing factors [7,8]. It may be hypothesized that alexithymia, acting on homeostatic mechanisms including the inner ear, could play an important role in the etiopathogenesis of the disease acting as a facilitating factor, as recognized in other medical conditions [11,27,28,29,30,31]. In our opinion, we are unable to assess the relationship between hydrops and alexithymia. We were unable to assess the relationship between MD and alexithymia. Although further studies are needed, it could be hypothesized that alexithymic personality trait may act as predisposing factor to develop hydrops, acting on neuroendocrine system. On the other side, more probably, alexithymic subjects are more prone to develop anxiety and depression, thus lowering their quality of life. For this reason this personality trait should be considered in order to decrease anxiety and depression of these subjects, thus increasing their quality of life.

This study has some limitations. First, only AMMI (Associazione Malati di Ménière Insieme) members were involved. Probably, patients who decided to join the Association perceive their disease as particularly impairing. It may be supposed that patients with less severe symptoms (e.g., a low number of vertigo spells) were less motivated to participate or were not included in the present research. Second, clinical features of MD were reported by patients themselves. We may consider these data quite reliable for statistical analyses. We would need, for future studies, a more systematic and accurate gathering of MD specific features. Third, our study had a cross-sectional design and, hence, did not allow causal inferences. Although this study has shown the importance of alexithymia in MD, we could not demonstrate the existence of a causality relationship between alexithymia and other psychological parameters in MD patients. Finally, the lack of a control group should be considered a limitation of this work.

As previously stated, further studies are needed to assess the true relationship between MD and alexithymia; the first target should be to increase the sample and compare data with those of a control group. For this purpose, we think that a multicentric study could be the best choice, and enrolled patients should be evaluated by an ENT specialist and a psychologist.

In conclusion, the present work represents a preliminary study that introduces a personality trait regarding emotion regulation and expression into the field of MD investigation. These considerations show the crucial importance of planning and possibly implementing a multidisciplinary clinical intervention for MD patients.

## Figures and Tables

**Table 1:** Clinical features of MD.

		N = 179
		Frequency (%)
Laterality	Left	69 (38.5%)
	Right	64 (35.8%)
	Bilateral	46 (25.7%)
Onset age	Mean (SD) y.o.	37.3 (11.7)
	Range	6–70
Number of crises in the last year	Mean (SD)	5.75 (8.1)
	Range	0–50

**Table 2:** Descriptive statistics of the psychological variables of the sample. The mean and standard deviation between parentheses, while in the second column the range are shown.

	Mean (SD)	Score Range
**TAS–20**		
DIF (difficulty in identifying feelings)	21.7 (6.7)	7–35
DDF (difficulty in describing feelings)	13.2 (4.4)	5–25
EOT (externally oriented thinking)	18.2 (4.5)	8–40
Total	53.2 (11.5)	20–100
**WHOQOL**		
Overall quality of life	2.97 (0.96)	1–5
General health	2.6 (0.92)	1–5
DOM 1: Physical health	52.64 (16.31)	0–100
DOM 2: Psychological	49.61 (13.11)	0–100
DOM 3: social relationships	55.27 (19.91)	0–100
DOM4: environment	55.72 (14.22)	0–100
**HADS**		
Anxiety	9.1 (4.2)	0–21
Depression	6.3 (4)	0–21
**PSS**		
Total stress	19.93 (7.1)	0–40
Perceived stress last week	5.74 (2.6)	0–10
Perceived stress last month	6.2 (2.6)	0–10
**COPE**		
Positive reinterpretation and growth	10.2 (2.85)	4–16
Mental disengagement	8.65 (2.2)	4–16
Focus on and venting of emotions	9.15 (2.37)	4–16
Use of instrumental social support	9.33 (3.17)	4–16
Active coping	10.49 (2.55)	4–16
Denial	5.9 (1.9)	4–16
Religious coping	8.4 (4.17)	4–16
Humor	7.35 (3.2)	4–16
Behavioral disengagement	6.86 (2.4)	4–16
Restraint	9.6 (2.6)	4–16
Use of emotional social support	8.9 (3.28)	4–16
Substance use	5.2 (2.2)	4–16
Acceptance	10.74 (3.16)	4–16
Suppression of competing activities	8.7 (2.3)	4–16
Planning	10.36 (2.9)	4–16

**Table 3:** Comparison between TAS-20 results in MD subjects and normative data by *t*-test expressed as mean and standard deviation between parentheses.

	MD	Normative Value	*t*-test
DIF (difficulty in identifying feelings)	21.7 (6.7)	14.6 (6)	T (178) = 14.3; *p* < 0.001
DDF (difficulty in describing feelings)	13.2 (4.4)	13.1 (4.8)	n.s.
EOT (externally oriented thinking)	18.2 (4.5)	17.1 (4.9)	T (178) = 3.3; *p* < 0.005
TOT	53.2 (11.5)	44.7 (11.3)	T (178) = 9.8; *p* < 0.001

**Table 4:** Age and Menière syndrome’s features comparison between A and NA groups by *t*-test.

	Non-Alexithymic	Alexithymic	
	M (sd)	M (sd)	*t*-test
Age	48.38 (11.32)	49.17 (11.71)	n.s.
Onset age	37.03 (11.12)	37.49 (12.18)	n.s.
Number of crises in the last year	4.77 (5.49)	6.64 (9.84)	n.s

**Table 5:** Psychological variables comparison between A and NA groups by *t*-test.

	Non-Alexithymic	Alexithymic	
	M (sd)	M (sd)	*t*-test
WHOQOL			
Overall quality of life	3.14 (0.94)	2.86 (0.97)	n.s
General health	2.66 (0.97)	2.55 (0.9)	n.s.
DOM 1: Physical health	55.36 (16.8)	50.71 (15.7)	n.s
DOM 2: Psychological	53.14 (13.1)	47.12 (12.6)	T (177) = 3.09; *p* < 0.005
DOM 3: social relationships	61.39 (20.2)	50.96 (18.6)	T (177) = 3.56; *p* < 0.001
DOM4: environment	57.7 (16.05)	54.29 (12.7)	n.s
HADS			
Anxiety	7.42 (3.8)	10.27 (4.2)	T (177) = −4.68; *p* < 0.001
Depression	4.54 (3.6)	7.58 (3.9)	T (177) = −5.31; *p* < 0.001
PSS			
Total stress	17.1 (6.4)	21.9 (6.9)	T (151) = −4.37; *p* < 0.001
Perceived stress last week	5.16 (2.9)	6.13 (2.3)	T (151) = −2.31; *p* < 0.05
Perceived stress last month	5.44 (2.7)	6.73 (2.3)	T (151) = −3.12; *p* < 0.005
COPE			
Positive reinterpretation and growth	11.5 (2.6)	9.33 (2.7)	T (157) = 5.08; *p* < 0.001
Mental disengagement	8.5 (2.5)	8.8 (1.9)	n.s.
Focus on and venting of emotions	9.5 (2.5)	8.9 (2.2)	n.s.
Use of instrumental social support	10.1 (3.2)	8.8 (3)	T (157) = 2.69; *p* < 0.01
Active coping	11.4 (2.4)	9.9 (2.5)	T (157) = 4.02; *p* < 0.001
Denial	5.6 (2)	6.1 (1.9)	n.s.
Religious coping	8 (4.1)	8.6 (4.2)	n.s.
Humor	8 (3.6)	6.9 (2.8)	T (157) = 2.24; *p* < 0.05
Behavioral disengagement	6.1 (1.9)	7.4 (2.6)	T (157) = −3.3; *p* < 0.005
Restraint	9.6 (2.8)	9.6 (2.6)	n.s.
Use of emotional social support	9.8 (3.4)	8.3 (3.1)	T (157) = 2.95; *p* < 0.005
Substance use	5 (2)	5.3 (2.4)	n.s.
Acceptance	11.7 (3.2)	10.1 (3)	T (157) = 3.1; *p* < 0.005
Suppression of competing activities	8.8 (2.4)	8.6 (2.2)	n.s.
Planning	11.5 (2.8)	9.6 (2.8)	T (157) = 4.17; *p* < 0.001

## Data Availability

We certify that this work was not previously send to other journals.
